# A conserved sequence in calmodulin regulated spectrin-associated protein 1 links its interaction with spectrin and calmodulin to neurite outgrowth

**DOI:** 10.1111/jnc.12462

**Published:** 2013-10-24

**Authors:** Mikayala D A King, Gareth W Phillips, Paola A Bignone, Nandini V L Hayes, Jennifer C Pinder, Anthony J Baines

**Affiliations:** * Department of Biosciences, University of KentKent, UK; † Randall Centre for Molecular Mechanisms of Cell Function, King's College LondonLondon, UK

**Keywords:** cytoskeletal regulation, cytoskeleton, molecular evolution, nerve cells, protein domain, protein function

## Abstract

Calmodulin regulated spectrin-associated protein 1 (CAMSAP1) is a vertebrate microtubule-binding protein, and a representative of a family of cytoskeletal proteins that arose with animals. We reported previously that the central region of the protein, which contains no recognized functional domain, inhibited neurite outgrowth when over-expressed in PC12 cells [Baines *et al*., *Mol. Biol. Evol*. **26** (2009), p. 2005]. The CKK domain (DUF1781) binds microtubules and defines the CAMSAP/ssp4 family of animal proteins (Baines *et al*. 2009). In the central region, three short well-conserved regions are characteristic of CAMSAP-family members. One of these, CAMSAP-conserved region 1 (CC1), bound to both βIIΣ1-spectrin and Ca^2+^/calmodulin *in vitro*. The binding of Ca^2+^/calmodulin inhibited spectrin binding. Transient expression of CC1 in PC12 cells inhibited neurite outgrowth. siRNA knockdown of CAMSAP1 inhibited neurite outgrowth in PC12 cells or primary cerebellar granule cells: this could be rescued in PC12 cells by wild-type CAMSAP1-enhanced green fluorescent protein, but not by a CC1 mutant. We conclude that CC1 represents a functional region of CAMSAP1, which links spectrin-binding to neurite outgrowth.

Elucidating the mechanisms that underlie the production of nerve axons is of fundamental importance to understanding the development and maintenance of the nervous system. It is well established that many elements of the cytoskeleton are required for this process, particularly in stabilizing and extending newly formed axons. Among these are microtubules (Lafont *et al*. 1993) and proteins that interact with them as assembly factors (e.g. Gordon-Weeks 2004), motors (e.g. Ahmad *et al*. 2000; Hirokawa and Takemura 2004) or cross-linkers (e.g. Dehmelt and Halpain 2004). Recently, we characterised a novel protein, calmodulin regulated spectrin-associated protein 1 (CAMSAP1), as a microtubule-binding protein (Baines *et al*. 2009). CAMSAP1 is expressed in nerve cells and glia in the nervous system (Baines *et al*. 2009; Yamamoto *et al*. 2009). CAMSAP1 contains a C-terminal CKK (PFAM: PF08683) domain which binds microtubules, and which, when over-expressed in the model cell line PC12, inhibits the production of neurites in response to nerve growth factor (NGF) (Baines *et al*. 2009).

CAMSAP1 is a member of a family of proteins that arose in evolution with the animals. In particular, all animals with differentiated tissues appear to have CAMSAP-like proteins, but no non-animal organism, or animals without tissues such as *Trichoplax* or sponge seem to have such proteins (Baines *et al*. 2009). The fruit fly orthologue of CAMSAP1 is encoded by the gene *ssp4/*patronin. This gene is required for normal mitotic spindle formation in S2 cells, potentially indicating a conserved role for members of this family of proteins in microtubule function by protecting the minus end of microtubules (Goshima *et al*. 2007; Goodwin and Vale 2010). A vertebrate paralogue of CAMSAP1, nezha (KIAA1543/CAMSAP3) has been reported to anchor microtubules at adherens junctions, in a complex containing PLEKHA7 and the minus end-directed motor KIF3c (Meng *et al*. 2008). One further paralogue, CAMSAP1L1 has been linked by genome-wide association study to epilepsy (Guo *et al*. 2012).

A possible interaction with intermediate filaments has also been suggested for CAMSAP1, potentially indicating that CAMSAP1 is a broad-function cytoskeleton cross-linking protein (Yamamoto *et al*. 2009). In neural tissue, the gene is regulated by the transcription factor calcium-response factor (West 2011).

In addition to a potential role for the CKK domain in neurite outgrowth, we reported indications of a role for the central region of the protein too (Baines *et al*. 2009) (see Fig. 1a, for an overview of the structure of CAMSAP1). Over-expression of the central region inhibited neurite outgrowth by PC12 cells. This region lies between the N-terminal calponin homology domain and the C-terminal CKK domain, and it contains no recognized structural or functional domains. However, it contains a proline-rich region and three regions that are relatively well conserved in evolution: we term these CAMSAP-conserved regions 1–3 (CC1–CC3) (Baines *et al*. 2009) (see Fig. 1a). Here, we have investigated further potential roles for CAMSAP1 in the production of processes from PC12 cells. Our data indicate interactions of the first of the CC1 with both spectrin and Ca^2+^/calmodulin. Knockdown of CAMSAP1 revealed a requirement for this protein in the production of neurites from PC12 cells, and rescue experiments link the spectrin-binding activity to the requirement for CAMSAP1. The CC1 region is conserved in the CAMSAP family, potentially indicating functional conservation from the earliest metazoa.

**Figure 1:**
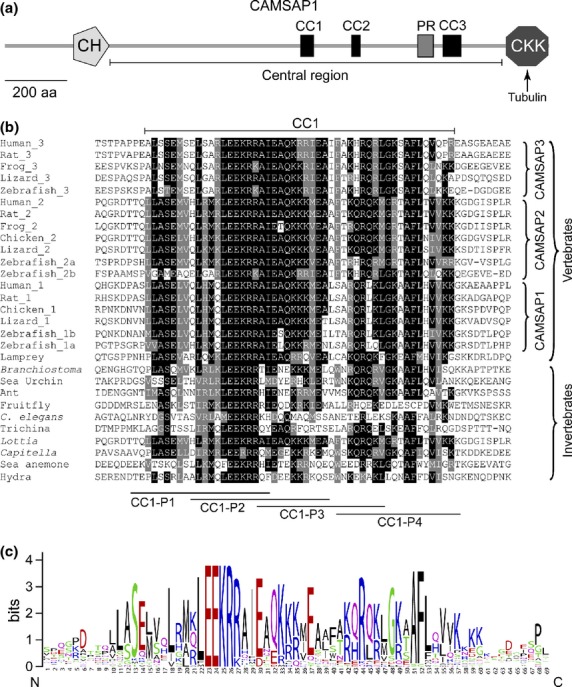
The CC1 region. (a) Domain structure of calmodulin regulated spectrin-associated protein 1 (CAMSAP1). CAMSAP1 has two known domains: a single CH domain near the N-terminus, and a CKK domain towards the C-terminus, which binds microtubules. The central region contains three conserved regions, CC1-3, and a proline-rich (PR) region. (b) Conservation of CC1 in members of the CAMSAP family. The figure shows alignment of the CC1 regions in a range of CAMSAP-family proteins across the metazoa. Sequences are listed in Table S1. The lines below the alignment labelled CC1-P1-4 show the locations of peptides synthesized to represent portions of CC1 (see Methods). (c) A sequence logo derived from the alignment in (b) above.

## Methods

### Antibodies

Antibodies to CAMSAP1 (Baines *et al*. 2009), brain spectrin (Hayes *et al*. 1997) and βIIΣ1-spectrin (Hayes *et al*. 2000) have been described previously. Monoclonal antibody to αII-spectrin D8B7 (Xu *et al*. 2000) was kindly given by Dr Leszek Kotula (New York Blood Center, New York, NY, USA). Anti-penta-his antibody was from Invitrogen (Renfrew, UK: P-21315). Anti-calmodulin was from Sigma-Aldrich (Poole, UK: C0931) as was anti-GAP-43 (G9264).

### Cell culture, transfection and siRNA methods

Culture methods for PC12 (Bignone *et al*. 2007; Baines *et al*. 2009) have been described previously: cells were grown on either laminin- or collagen-coated coverslips. Cultures of rat cerebellar granule cells have been described previously (Hayes *et al*. 2000). A human CAMSAP1-enhanced green fluorescent protein (EGFP) construct has been described previously (Baines *et al*. 2009). siRNA oligomers (485 siRNA for CAMSAP1 sense strand CGACACAGAUCGAAUUCUUUdTdT) were purchased from Eurofins MWG (Ebersberg, Germany), and were transfected using Fugene at a concentration of 5 nM. Cells were analysed by fluorescence microscopy (for EGFP fluorescence and by immunofluorescence) as described previously (Bignone *et al*. 2007) up to 6 days post-transfection.

### Proteins and peptides

Human CAMSAP1 cDNA has been described previously (Baines *et al*. 2009). A his-tagged construct of part of the central region of rat CAMSAP1 was expressed in *E. coli* from the vector pQE32 (Qiagen, Crawley, UK): the sequence of this cDNA is equivalent to residues 2157–4160 in Refseq:XM_001078134. It was purified from soluble extracts of *E. coli* BL21 DE3, pLysS by metal chelating chromatography. A his-tagged human CC1 region construct was expressed in *E. coli* from the vector pET15b (Novagen, Nottingham, UK): the sequence of this is equivalent to Uniprot:Q5T5Y3: [873–921]. It was purified from soluble extracts of *E. coli* Rosetta 2 (Merck, Nottingham, UK) by metal chelating chromatography. Glutathione-s-transferase (GST)-fusion proteins of human βIIΣI-spectrin fragment from repeat 16 to the C-terminus (R16-C), βIIΣ2-spectrin R16-C, βIIΣ1-spectrin linker have been described previously (Hayes *et al*. 2000). Recombinant peptides were verified by mass spectrometry. Brain spectrin (Bennett *et al*. 1986) and calmodulin (Dedman and Kaetzel 1983) were purified from pig brain by standard procedures. Four peptides representing overlapping fragments of CC1 were synthesized: CC1-P1 ASLLASELVQLHMQLEEKRRAI [equivalent to Uniprot: Q5T5Y3: (871–892)], CC1-P2 QLHMQLEEKRRAIEAQKKKMEA [Q5T5Y3: (880–901)], CC1-P3 RAIEAQKKKMEALSARQRLKL [Q5T5Y3: (890–910)], CC1-P4 SARQRLKLGKAAFLHVVKKG [Q5T5Y3: (903–922)]. A 29mer from the linker region of βIIΣ1-spectrin was also synthesized EMVNGATEQRTSSKESSPIPSPTSDRKAK [Uniprot: Q01082: (2149–2177)]. All peptides were made with an N-terminal biotin-hexanoic acid tag in-house, by Mr Kevin Howland (University of Kent, Biomolecular Sciences facility). They were purified and verified by HPLC and mass spectrometry.

### Interactions of spectrin

Brain spectrin, GST-fusion proteins of βIIΣI-spectrin R16-C (triple helical repeat 16 to the C-terminal), βIIΣ2-spectrin R16-C, βIIΣ1-spectrin linker, or his-tagged construct of CC1 were coupled to supporting media. (i) *Affinity chromatography*. Methods for interaction with the his-tagged CAMSAP1 central region construct were essentially as described elsewhere (Hayes *et al*. 1997). Briefly, for affinity chromatography, the CAMSAP1 fragment (10 μg/mL) was chromatographed on 0.5 mL of individual spectrin proteins coupled to Activated CH-Sepharose 4B (GE Life Sciences, Amersham, UK), or control columns consisting of bovine serum albumin coupled to Sepharose, or plain Sepharose 4B. The running solution was 0.15 M NaCl, 10 mM HEPES/NaOH pH7.4, with 1 mM EGTA or 1 mM CaCl_2_ as indicated in the text. The columns were washed with 10 column volumes of running solution. Bound material was recovered by washing with 1 M KI, 10 mM HEPES/NaOH pH7.4. Bound fractions were analysed by sodium dodecyl sulfate (SDS) gel electrophoresis and immunoblot with anti-penta-his antibody. (ii) *Pull down assays*. For pull-down experiments, 100 μL (packed volume) of his-tagged CC1 coupled to CH-Sepharose 4B was mixed with a cytosolic preparation from rat brain in 0.32 M sucrose, 10 mM HEPES/NaOH pH7.4, 5 mM EGTA and incubated at 4° for 60 min. The reaction mixture (500 μL) was placed in a MicroSpin column (Amersham-Pharmacia Biosciences, GE Healthcare Lifesciences, Buckinghamshire, UK) and unbound proteins were removed by centrifugation at 750 g for 1 min. The Sepharose was washed three times with 0.32 M sucrose, 10 mM HEPES/NaOH pH7.4, 5 mM EGTA, 0.1% Triton X100. Bound proteins were eluted with 60 μL of 2 × Laemmli sample buffer (Laemmli 1970). Samples were boiled for 5 min and separated by SDS gel electrophoresis and analysed by immunoblotting with anti-brain spectrin. In control reactions, blanks with uncoupled Sepharose 4B or no brain extract were also run. In some cases, competing peptides were added to 10 μg/mL. Samples were analysed by immunoblotting. Immunoblots were developed with enhanced chemiluminescence (Amersham-Pharmacia Biosciences) reagents. (iii) *Micro-titre plate assays*. His-tagged CC1 was immobilized on the wells of a 96 well polystyrene plate by incubating 50 μL of a 10 μg/mL solution of CC1 in 0.1 M NaCO_3_ at 4° overnight. The plates were washed four times with 0.15 M NaCl, pH 7.4 and residual protein binding capacity blocked by incubating the wells with 10 mg/mL bovine serum albumin for 1 h. The plates were washed again four times, and spectrin 29mer peptide was incubated in the plates for 2 h at 23°C. Unbound material was removed by washing five times in buffer, and bound peptide was detected by incubating the plates with Streptavidin-peroxidase (3 μg/mL) for 30 min, followed by five more washes and colour development with o-phenylenediamine. (iv) *Immunoprecipitation from brain extract*. Methods for immunoprecipitation have been described previously (Scott *et al*. 2001). Briefly, anti-CAMSAP1 IgG or control IgG was used at 10 μg/mL final concentration. IgG were mixed with brain extract (as above) for 1 h on ice, in the presence of Ca^2+^, EGTA or trifluoperazine (TFP) as noted in the text. IgG with bound material were recovered by adding Protein A beads (Amersham-Pharmacia Biosciences), and removing unbound material by spinning over Micro-Spin columns and washing three times. Bound proteins were eluted with 60 μL of 2 × Laemmli sample buffer (Laemmli 1970). Samples were boiled for 5 min and separated by SDS gel electrophoresis, followed by immunoblotting with antibodies as noted in the text.

### Interactions of calmodulin

Calmodulin was labelled with biotin using biotin-hexanoic acid-N-hydroxysuccinimide (Sigma, Poole, UK) as described previously (Nicol *et al*. 1997). (i) *BIAcore*. Interactions of calmodulin were measured using BIAcore analysis as previously described (Nicol *et al*. 1997). (ii) *Peptide pull down*. To assay interaction of synthetic CAMSAP1 peptides, except as noted in the text, biotinylated peptide CC1-P4 (see above for sequence, 10 μg/mL) was mixed with a cytosolic preparation from rat brain in 0.32 M sucrose, 10 mM HEPES/NaOH pH7.4 containing 1 mM CaCl_2_ or 5 mM EGTA and incubated at 4° for 60 min. The reaction mixture (500 μL) was mixed in a MicroSpin column (Amersham-Pharmacia Biosciences) with 60 μL of a 25% slurry of streptavidin–Sepharose equilibrated in binding buffer, and incubated for 60 min. Unbound proteins were recovered by centrifugation at 750 *g* for 1 min. The Sepharose was washed three times with binding buffer. Bound proteins were eluted with 60 μL of 2 × Laemmli sample buffer (Laemmli 1970). Samples were boiled for 5 min and separated by SDS gel electrophoresis. In control reactions, blanks with no peptide or no brain extract were also run. Samples were analysed by immunoblotting and probing with anti-calmodulin.

### Bioinformatics

BLAST searches were run using NCBI BLAST (Altschul *et al*. 1997) installed locally. A hidden Markov model for CC1 was prepared from alignments initially generated by BLAST and used to interrogate the NCBI nr (Sayers *et al*. 2009) and Uniprot Knowledgebase (Bairoch *et al*. 2005) databases, using HMMer (Eddy 2011) installed locally. Potential calmodulin binding sites were identified using the Calmodulin Target Database (Yap *et al*. 2000).

### Statistical analysis of neurite outgrowth

Neurite production by PC12 cells was scored on the basis that cell processes extended for more than the diameter of the cell body. Experiments described here represent neurite counts from three separate transfections, each divided onto three to five coverslips. Counts were made from each coverslip and recorded separately. Neurite scores were pooled to give 9–15 individual measurements, with approximately 150–250 cells counted per condition. For cerebellar granule cells, the criterion for axon growth was GAP43-positive processes extended for more than twice the cell body. Data are presented as mean ± SD, and were analysed using Student's *t*-test. In the figures, * indicates *p* < 0.01; **p < 0.001 and ***p < 0.0001.

## Results

### The CC1 region is highly conserved in the CAMSAP family

Figure 1a, shows a schematic diagram of the domain structure of human CAMSAP1. The characteristic domains are a calponin homology (CH) domain and a microtubule-binding CKK domain close to the C-terminus. The combination of CH and CKK domains defines the CAMSAP family. In addition, the central region of the protein, when over-expressed in PC12 cells, inhibited neurite outgrowth (2009), so this region attracted our attention in relation to possible function. Within the central region, alignment of several CAMSAP family proteins revealed three relatively well-conserved parts (CAMSAP-conserved 1–3, CC1–3 residues 873–921, 1015–1051 and 1262–1359 respectively in Uniprot: Q5T5Y3) (see Fig. 1 in Baines *et al*. 2009).

Figure 1b, shows an alignment of the CC1 region from a variety of eumetazoan organisms. CC1 is a region of about 50 residues with high conservation surrounded by sequence that shows little-to-no conservation. Fig. 1c represents the alignment as a sequence logo: the sequence LEEKR highlighted in the logo is conserved between hydra and man.

CC1 appears to have arisen early in metazoan evolution: as shown in Fig. 1b, examples exist in deuterostomia (e.g. the chordates and sea urchin *Strongylocentrotus purpuratus*) ecdysozoa (arthropods and nematodes), cnidaria (hydrazoan *Hydra magnipapillata* and anthozoan sea anemone *Nematostella vectensis*) and lophotrochozoa (segmented worm *Capitella teleta* and limpet *Lottia gigantea*).

Human CAMSAP1 CC1 is 60% identical to human CAMSAP2 CC1 and 43% identical to human CAMSAP3 CC1, as measured using the Needle program in EMBOSS (Rice *et al*. 2000). It is also 62% identical to its orthologous sequence in zebrafish; 49% identical to *Branchiostoma* CC1; 32% identical (52% similar) to the sea anemone sequence and 32% identical (51% similar) to sea urchin. Ecdysozoa have diverged further: human CAMSAP1 CC1 is 27% identical (37.3% similar) to fruitfly CC1; *C. elegans* CC1 is more similar to human CAMSAP2 CC1 than that of human CAMSAPs 1 or 2, 33% identical (51% similar).

The only exception we have found to the broad expression of CC1 in eumetazoa is in acoelomata. The genomic coverage of these is limited as yet, but we were unable to detect CC1 in the genome of *Schistosoma mansoni* either by Blast analysis of genomic sequence or HMM search of predicted peptides. Blast interrogation of acoelomate expressed sequence tags further revealed no candidate CC1 sequences. One possibility is that CC1 was lost in the evolution of at least some acoelomates.

### CAMSAP1 interacts with spectrin

In preliminary experiments (data not shown) we tested possible interactions of the central region of CAMSAP1 by affinity chromatography of brain extracts on the immobilized central region. Immunoblots of the resulting bound fractions revealed brain spectrin as a binding partner. Spectrin is an elongated F-actin cross-linking protein and it is an α_2_β_2_ heterotetramer. It has many binding partners including other cytoskeletal proteins, membrane proteins and regulatory proteins (Baines 2010b). To characterize potential CAMSAP1-spectrin interaction further, we have used spectrin purified from brain (a mixture of isoforms, but primarily αII/βII-spectrin), as well as recombinant fragments.

**Figure 2:**
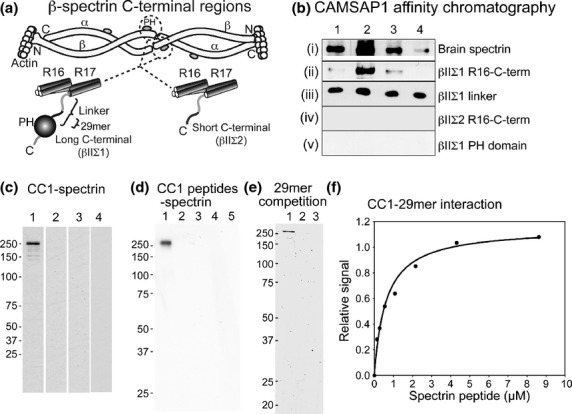
Interaction of calmodulin regulated spectrin-associated protein 1 (CAMSAP1) with spectrin. (a) Generalized structures of spectrin and C-terminal variants of β-spectrins. Spectrin α and β subunits are arranged in tetramers. At the C-terminal end of β chains, splice variation gives rise to long (βIIΣI) and short (βIIΣ2) variants. The long variant has a PH domain joined to the last of the helical repeats (repeats 16 and 17) by a linker of around 90 amino acids. Differential mRNA splicing gives rise to a variant in which this linker is interrupted such that the PH domain is lost and the second half of the linker is replaced with a unique (probably unstructured) region in the short variant. (b) Affinity chromatography of CAMSAP1 on various spectrin constructs coupled to Sepharose. A his-tagged central region construct (see Fig 1a) was flowed over the indicated spectrin protein or fragment coupled to Sepharose. Unbound material was washed away, and bound material recovered by elution with 1 M KI. Fractions representing bound material were analysed by immunoblot: sequential fractions from the 1M KI elution, labelled 1–4, are shown here. Note that the CAMSAP1 fragment bound to whole brain spectrin, the βIIΣI C-terminal fragment from repeat 16 to the C-terminus, the βIIΣI linker fragment, but not to the fragment of βIIΣ2 repeat 16 to C-terminus or the PH domain alone. (c) Interaction of brain spectrin with the CAMSAP-conserved region 1 (CC1) region. His-tagged CC1 construct was coupled to Sepaharose and mixed with brain extract. The figure shows an immunoblot of material recovered after washing the beads in buffer and elution with sodium dodecyl sulfate (SDS). Lanes 1-4 show immunoblots probed with antibody to brain spectrin. Lane 1: CC1-Sepharose. Lane 2: bovine serum albumin coupled to Sepharose. Lane 3: Unconjugated Sepharose. Lane 4: AAAA mutant. (d) Interaction of peptides from CC1 with spectrin. Biotinylated peptides CC1-P1-4 (see Methods and Fig 1b) were mixed with brain extract and recovered on streptavidin-Sepharose. Bound material was eluted with SDS and analysed by immunoblotting with anti-brain spectrin. Lane 1, CC1-P1; Lane 2, CC1-P2; Lane 3, CC1-P3; Lane 4, CC1-P4; Lane 5, no peptide. Note that spectrin bound to Pep1. (e) Competition between a 29mer peptide representing part of the linker region from βIIΣI and brain spectrin. CC1-Sepharose was mixed with brain extract in the absence (Lane 1) or presence (Lane 2) of the 29mer. Bound material was recovered and analysed by immunoblotting with anti-brain spectrin. Note that in the absence of peptide, spectrin was recovered, but that in the presence of peptide no spectrin was recovered. Lane 3 shows a control with CC1-Sepharose processed as in lane 1, but without added brain extract. (f) Binding between the spectrin 29mer and CC1. His-tagged CC1 was immobilised in 96-well plates and incubated with biotinylated 29mer. Binding was detected using streptavidin-peroxidase. The curve is calculated for a single class of binding site, *K*_D_ 650 nM.

Figure 2a, summarizes the structure of αII/βII-spectrin tetramers: note that there are two C-terminal splice variants of βII-spectrin. A long C-terminal variant (βIIΣ1) contains a pleckstrin homology domain joined to the last of the helical repeats (the partial repeat 17) via a linker region. A short splice (βIIΣ2) variant arises from differential mRNA splicing that eliminates the PH domain and part of the linker region.

Figure 2b, shows analysis of the interaction of CAMSAP1 central region with brain spectrin and its fragments by affinity chromatography. For this, we used a 746 amino acid fragment of rat CAMSAP1: this contains CC1-3 and the proline-rich region, but not the recognized CH and CKK domains. Brain spectrin or its fragments were coupled to activated Sepharose 4B. For affinity chromatography, the CAMSAP1 fragment was flowed over columns of the coupled spectrin proteins; the columns were then washed and bound material was recovered by elution with 1M KI. Fig. 2b shows material recovered in KI elutions.

Figure 2bi, shows that the CAMSAP1 fragment was recovered in the fractions representing material that had bound to the whole brain spectrin column. Columns prepared from a fragment of the long C-terminal variant of βII-spectrin (GST-βIIΣI-R16-C) also retained CAMSAP1 on the column (Fig. 2bii), as did the whole linker region from the long C-terminal variant fused to the GST (Fig. 2biii). In contrast, a fragment representing repeat 16 to the C-terminus of the short C-terminal variant (βIIΣ2) fused to GST did not retain CAMSAP1 fragment on the column (Fig. 2biv). Furthermore, a construct representing the βII-spectrin PH domain coupled to GST did not retain the CAMSAP1 fragment (Fig. 2bv). GST-Sepharose (data not shown) also did not retain it on the column. These data indicate that CAMSAP1 interacts with the linker region specific to the long C-terminal variant of βII-spectrin.

To identify the site in CAMSAP1 that binds spectrin, we tested a number of fragments of CAMSAP1 for their ability to bind spectrin. Neither the CH or CKK domains showed significant interaction (data not shown). To probe the central region further, CC1 was expressed as a his-tagged polypeptide in bacteria, coupled to activated Sepharose, and tested for its ability to bind to brain spectrin in a whole brain extract (Fig. 2c). Brain spectrin was readily detected in immunoblots of the recovered bound material (Fig. 2c, Lane 1). No spectrin was recovered from beads coupled to bovine serum albumin (Lane 2), or (Lane 3) plain Sepharose beads. A mutant CC1 was prepared in which the characteristic LEEK sequence was replaced by AAAA: this showed no interaction with spectrin (Lane 4).

The CC1 region is quite short, so to define the nature of binding more closely, four overlapping biotinylated peptides covering the whole of CC1 were synthesized (Peptides CC1-P1-4, see Methods and Fig. 1b). These were tested for their ability to bind spectrin in whole brain extract. In a pull-down assay (Fig. 2d), only CC1-P1 bound to spectrin, thus the spectrin binding activity is associated with the N-terminal part of CC1.

The βII-spectrin linker region between the last helical repeat and the PH domain is differentially spliced (see Fig. 2a): the affinity chromatography experiments indicated in Fig. 2b implicate this sequence in CAMSAP1 binding. The part of the linker specific to the long form (βIIΣ1) is encoded by a single exon (ENSEMBL:ENSE00001306451) (Hayes *et al*. 2000). Blast analysis of this in comparison to other vertebrate βIIΣ1-spectrins revealed 29 amino acids were well conserved [Uniprot: Q01082: (2149–2177)], so we chose these for analysis. A peptide representing this 29 amino acid sequence was synthesized. This was used to compete with brain spectrin in a pull-down experiment. CC1-Sepharose beads were mixed with brain extract in the absence (Fig. 2e, Lane 1) or presence (Lane 2) of the peptide. Note that no brain spectrin is recovered in the presence of the peptide. Lane 3 shows a control in which no brain extract was added to plain Sepharose beads.

Figure 2f, shows quantification of the binding of the CC1 region to the 29mer peptide. For this, CC1 was coated in the wells of a 96-well plate and increasing concentrations of the biotinylated peptide were added; binding was detected by adding streptavidin peroxidase followed by colorimetric detection. The data are consistent with a single class of binding site, as shown by the curve, with a *K*_D_ 650 nM.

We conclude that CAMSAP1 interacts with spectrin via its CC1 region, and that the binding site in spectrin lies within a 29 amino acid sequence adjacent to the PH domain of the long C-terminal variant of βII spectrin.

### CAMSAP1 interacts with calmodulin

In analysing the sequence of CAMSAP1 (and other members of the family) we noted the presence of a potential calmodulin binding sequence within the CC1 region (residues 903-922 in Uniprot: Q5T5Y3) predicted by the Calmodulin Target Database (Yap *et al*. 2000). Thus, we investigated whether CAMSAP1 could bind calmodulin.

**Figure 3:**
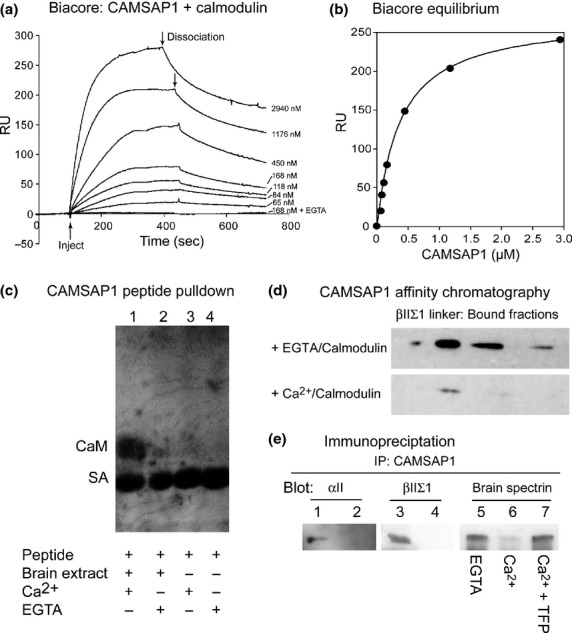
Calmodulin regulates the interaction of calmodulin regulated spectrin-associated protein 1 (CAMSAP1) with spectrin. (a) Biacore analysis. Biotinylated calmodulin was captured on a streptavidin Biacore chip, and the central fragment of CAMSAP1 flowed over it at various concentrations, and in the presence of Ca^2+^, or EGTA as indicated. Binding was detected as in increase in Resonance Units (RU) from the instrument. Note that no binding occurs in EGTA. At the points marked by arrows, the CAMSAP1 flow was halted and buffer only was flowed over the chip to allow dissociation to occur. (b) Equilibrium values for binding. From (a) equilibrium values for binding (in RU) were calculated for each concentration. The curve shown is calculated for *K*_D_ 620 nM. (c) Interaction of a peptide from CAMSAP-conserved region 1 (CC1) with calmodulin. Biotinylated CC1-P4 (see Methods) was mixed with brain extract in the presence and absence of Ca^2+^ as indicated. Peptides were recovered on strepatavidin-Sepharose and analysed by immunoblot with anti-calmodulin. Note that strong reactivity with calmodulin was only detected in the presence of Ca^2+^. The strong band in all lanes represents streptavidin eluted from the beads in sodium dodecyl sulfate (SDS): it reacts strongly with the secondary antibody. (d) Affinity chromatography of CAMSAP1 central fragment on GST-βIIΣI-spectrin linker coupled to Sepharose in the presence of calmodulin and either EGTA or Ca^2+^ as indicated. The figure shows fractions from the column after elution with KI, representing material that had bound to the column. Note that strong reactivity with calmodulin was only detected in the presence of Ca^2+^. (e) Immunoprecipitation of CAMSAP1 and spectrin. Brain extract was analysed using immunoprecipitation with anti-CAMSAP1 antibody. Lanes 1 and 2 show material analysed by immunoblotting with anti-αII-spectrin: Lane 1, anti-CAMSAP1 immunoprecipitate; Lane 2 control. Lanes 3 and 4 were probed with anti-βIIΣI antibody: lane 3 anti-CAMSAP1 immunoprecipitate; Lane 4 control. Lanes 5-7 were all probed with anti-brain spectrin: Lane 5 immunoprecipitation of CAMSAP1 in the presence of EGTA; Lane 6, in the presence of Ca^2+^; Lane 7, with Ca^2+^ and the calmodulin inhibitor trifluoperazine (100 μM). Note that lanes 5–7 show a small amount of a spectrin proteolytic product at the bottom of the lanes.

Figure 3a, shows analysis of CAMSAP1-calmodulin interaction using surface plasmon resonance (BIAcore). The central region his-tagged fragment of CAMSAP1 was tested for its ability to bind to biotinylated calmodulin immobilised on a streptavidin-coupled CM5 BIAcore chip. The data indicate that CAMSAP1 interacts with the chip, and that the binding is completely dependent on calcium, since little or no signal is obtained when CAMSAP1 is flowed onto the chip in the presence of EGTA. To quantify the binding, equilibrium values were calculated for each concentration of CAMSAP1 used; the data are plotted in Fig. 3b, which fit a calculated curve for *K*_D_ 620 nM.

The potential calmodulin-binding site was contained within synthetic peptide CC1-P4 (see Methods and Fig. 1b). The biotinylated peptide was added to brain extract in the presence of either calcium or EGTA and the peptide was recovered using streptavidin beads (Fig. 3c). Lane 1 shows an immunoblot of material recovered in the presence of calcium probed with an anti-calmodulin antibody: note that calmodulin is detected. In contrast, little to no calmodulin was recovered in the presence of EGTA (Lane 2). Lanes 3 and 4 show corresponding material recovered in the absence of added brain extract.

Since both spectrin and calmodulin bound to the CC1 region, we tested whether calmodulin could alter the binding of spectrin. Using affinity chromatography, the his-tagged central fragment of CAMSAP1 was mixed with calmodulin in the presence or absence of Ca^2+^, and chromatographed the mixtures on GST-βIIΣI-R16-C Sepharose (Fig. 3d). Bound material was analysed by anti-his tag immunoblot. Bound fractions were found to contain the CAMSAP1 fragment in the presence of EGTA, but not in the presence of Ca^2+^. These data indicate that Ca^2+^/calmodulin negatively regulates the interaction of CAMSAP1 with spectrin.

To test if a similar modulation of the interaction might occur in more complex mixtures, we used immunoprecipitation from a brain extract. Whole brain extract was immunoprecipitated using CAMSAP1 antibodies under non-denaturing conditions. The immunoblots were probed with either antibodies to αII-spectrin (Fig 3e, lane 1) or the long C-terminal variant of βII-spectrin (Lane 3). Both of these polypeptides were found in the immunoprecipitates, and not in non-immune IgG controls (Lanes 2 and 4). To examine if the binding was dependent on active calmodulin, we repeated the immunoprecipitation in the presence of EGTA (lane 5), calcium (Lane 6) or calcium plus the calmodulin inhibitor TFP. Note that spectrin (detected with antibody to whole brain spectrin) is recovered in the immunoprecipitate in the presence of EGTA but not calcium. Immunoprecipitation is restored by inhibition of calcium/calmodulin with TFP. These data indicate that both spectrin and calmodulin bind to the CC1 region, and that calmodulin negatively regulates the interaction of spectrin.

### Over-expression of CC1 in PC12 cells inhibits neurite outgrowth

Previously, we showed that the central region of CAMSAP1 inhibited neurite outgrowth from PC12 cells (Baines *et al*. 2009). Since CC1 appears to be a site of protein-protein interaction, we tested whether it had any effect on neurite extension, under the same conditions we used previously.

EGFP-CC1 was transfected into PC12 cells, and neurite production was induced by NGF treatment (see Methods). After 48 h, the proportion of green fluorescent cells that showed neurite production was counted. The results are summarized in Table 1. Note that cells transfected with EGFP alone yielded 76 ± 11% with neurites. EGFP-CC1 reduced the proportion of cells developing neurites to 42 ± 17%. For comparison, we also transfected the AAAA mutant of CC1: this showed no significant inhibition of neurite outgrowth (69 ± 14%).

**Table 1:** Effect of over-expression of CAMSAP1 CC1 on neurite extension by PC12 cells

Construct	% fluorescent PC12 Cells with neurites ± SD	
EGFP alone	76 ± 11	
EGFP-CC1	42 ± 17	*p *=* *2 × 10^−5^
EGFP-CC1-AAAA	69 ± 14	*p *= NS

PC12 cells were transfected with the indicated construct and neurite extension was induced with nerve growth factor. Neurite production was scored as described in Bignone *et al*. (2007).

### siRNA reveals a requirement for CAMSAP1 in neurite outgrowth

**Figure 4:**
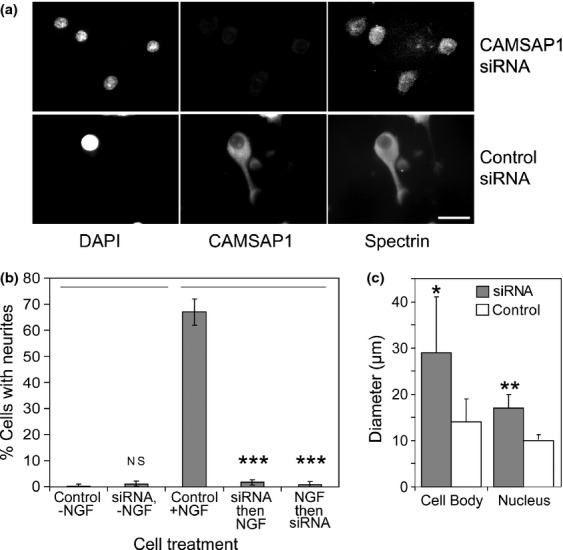
siRNA analysis of calmodulin regulated spectrin-associated protein 1 (CAMSAP1) function in PC12 cells. (a) PC12 cells were transfected with either rat CAMSAP1 siRNA or control, scrambled siRNA, and treated with nerve growth factor (NGF). Cells were stained after 48 h, as indicated, with either DAPI (for DNA), anti-CAMSAP1 or anti-βIIΣ1-spectrin. Note reduction in the staining of CAMSAP1 compared to cells treated with negative control siRNA. βIIΣ1-spectrin staining is not affected, indicating that CAMSAP1 is not required for accumulation of spectrin. The spectrin staining shows that the CAMSAP1 knock down cells have failed to extend neurites. Cells transfected with control siRNA extended neurites. Scale bar 10 μm. (b) Quantification of% cells with neurites. Comparing cells treated with control siRNA or CAMSAP1 siRNA in the absence of NGF showed no significant difference (NS). However, with NGF, the reduction of neurite outgrowth was highly significant with CAMSAP1 siRNA in comparison with control siRNA. (c) Quantification of cell and nuclear diameters.

To test a requirement for native CAMSAP1 in neurite outgrowth, we used siRNA to knock down the expression of CAMSAP1 in PC12 cells. Fig. 4a–c, show the result of transfecting PC12 cells with siRNA designed to be specific for rat CAMSAP1. Fig. 4a, shows fluorescence microscopy in NGF-treated CAMSAP1 siRNA-transfected cells (upper panel) and control siRNA (lower panel). Note that while CAMSAP1 immunofluorescence was detectable in the control treated cells, it was essentially undetectable in the specific CAMSAP1 treated cells. DAPI (4′,6-Diamidino-2-Phenylindole) (DNA) and spectrin stains are retained in these cells, as in the controls, so CAMSAP1 is not required for spectrin accumulation in PC12 cells. Strikingly, however, no neurites are visible in the CAMSAP1 siRNA-transfected cells (anti-spectrin stain), unlike controls.

This effect of CAMSAP1 siRNA was quantified as described by microscopy (Fig. 4b). Cells treated with control siRNA produced essentially no neurites in the absence of NGF, but efficiently responded to NGF by producing neurites. However, in the presence of NGF and CAMSAP1 siRNA fewer than 5% of transfected cells produced neurites over 2–4 days. It made no difference whether NGF was added an hour before or after transfection with siRNA.

It was also evident in microscopy that after over the same period, CAMSAP1 siRNA-transfected cells appeared larger than controls (Fig. 4c). Both cell diameter and nuclear diameter increased.

**Figure 5:**
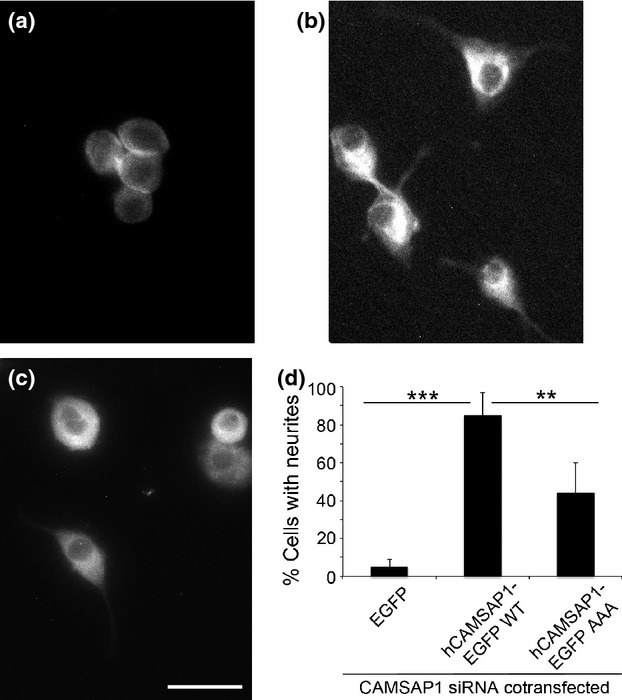
Rescue of calmodulin regulated spectrin-associated protein 1 (CAMSAP1) siRNA knockdown in PC12 cells. PC12 cells were transfected with CAMSAP1 siRNA, simultaneously with vectors encoding (a) enhanced green fluorescent protein (EGFP) alone (b) human CAMSAP1-EGFP wild type or (c) human CAMSAP1-EGFP AAAA mutant. Scale bar 20 μm. The figure shows EGFP fluorescence images. Cells expressing EGFP were identified scored for the production of neurites. Quantification of neurite production in each case is summarised in (d).

As a further control for the specificity of the siRNA, we attempted to rescue the knockdown of the rat CAMSAP1 with human cDNA. To identify cells transfected with both siRNA and EGFP constructs, we took advantage of the nature of the anti-CAMSAP1 antibody: since this is an antibody to the C-terminal peptide, reactivity is blocked by fusion to EGFP. Cells were scored for siRNA transfection by loss of endogenous CAMSAP1 immunofluorescence and for EGFP expression, also by fluorescence. Human CAMSAP1 differs at two positions in the sequence recognized by the siRNA. This should make it resistant to the siRNA. Accordingly, PC12 cells were transfected with CAMSAP1 siRNA and either (Fig. 5a) EGFP alone or (Fig. 5b) human CAMSAP1-EGFP. Neurites were produced from the hCAMSAP1-EGFP transfected cells indicating a rescue.

The rescue also provided the opportunity to test the significance of the conserved LEEK motif in CC1 identified in Figs 3. A mutant CAMSAP1-EGFP was prepared in which the conserved LEEK motif was changed to AAAA (Fig. 5c): a lower proportion of cells were able to produce neurites when transfected with the construct. Quantification of the rescues (Fig. 5d) revealed that few neurites were produced from cells co-transfected with siRNA and EGFP alone, but native hCAMSAP1-EGFP gave an efficient rescue (85 ± 11% neurite production). The AAAA mutant gave a substantially weaker rescue: 37 ± 16%. Collectively, these data indicate that CAMSAP1 is required for NGF-induced neurite production in PC12 cells, and that the LEEK motif in CC1 that links CAMSAP1 to spectrin has a role in this process.

### CAMSAP1 siRNA inhibits axon production in primary granule cell cultures

**Figure 6:**
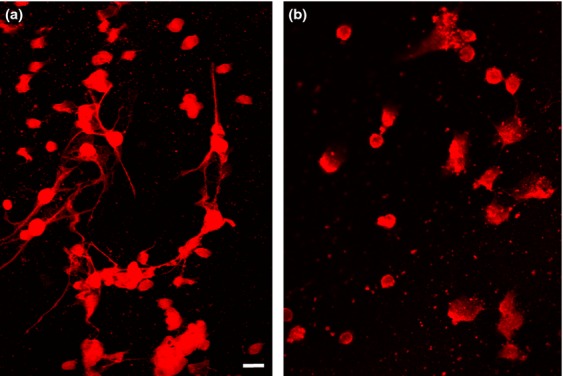
siRNA knockdown of calmodulin regulated spectrin-associated protein 1 (CAMSAP1) in cerebellar granule cell cultures. Cultures of cerebellar granule cells were examined by immunofluorescence with anti-GAP43. (a) shows granule cells transfected at the time of isolation with control siRNA; (b) shows cells transfected with CAMSAP1 siRNA. Note the absence of extended GAP-43 positive processes in the siRNA-treated cells, although such processes are readily seen in control cells. Scale bar 20 μm.

We wished to confirm the significance of CAMSAP1 in the growth of neuronal processes by examining primary cells, in addition to the PC12 cell line. For this purpose we cultured cerebellar granule cells as described previously (Hayes *et al*. 2000). They were transfected with control (Fig. 6a) or CAMSAP1 siRNAs (Fig. 6b) and examined by immunofluorescence using antibody to GAP43. Axon processes were readily detected in samples transfected with control siRNA, but were essentially absent from those transfected with the specific CAMSAP1 siRNA. In three separate preparations of cells, less than 2% of cells transfected with CAMSAP1 siRNA carried GAP43-positive processes longer than twice the diameter of the cell body. In contrast, about a quarter of cells in each culture transfected with control siRNA carried such processes (24 ± 11%, *p* < 0.0001)

## Discussion

In this report, we have provided evidence that CAMSAP1 is required for the process of neurite production by PC12 cells (Figs 4 and 5, Table 1) and primary cerebellar granule cells (Fig. 6). Moreover, we define interactions with spectrin and Ca^2+^/calmodulin (Figs 3) that indicate that CAMSAP1 is a regulated cytoskeletal interconnector, and that CC1 is a novel functional domain containing these activities.

Our earlier data pointed to activities in CAMSAP1 additional to microtubule binding (Baines *et al*. 2009), since over-expression of the central region also inhibited PC12 cell neurite outgrowth. This region lacks previously recognized functional domains and formed a rather diffuse pattern when over-expressed in HeLa cells, reminiscent of the pattern described for spectrin (Mangeat and Burridge 1983). Over-expression of active fragments of CAMSAP1, the CKK domain or the whole central region (Baines *et al*. 2009), and, as shown here, the CC1 region, should allow them to act as inhibitors that compete with the native protein for its physiological binding sites and disrupt function as a cytoskeletal interconnector.

We tested an interaction with spectrin using affinity chromatography, in the first instance, with a fragment of rat CAMSAP1 corresponding to 668 residues of the central region. The data indicated an interaction selectively with the linker region between the last of the triple helices and the PH domain (Fig. 2b). In peptide binding experiments, this was narrowed to 29 residues unique to the long C-terminal form of βII-spectrin (βIIΣ1) (Fig. 2c and d). In turn, this peptide bound to the CC1 region in CAMSAP1 (Fig. 2e). The 29mer sequence comes from a region of spectrin to which no closely defined function has previously been ascribed. PSI-PRED (McGuffin *et al*. 2000) and DISO-PRED (Ward *et al*. 2004) analyses (data not shown) indicate this part of spectrin is likely to be almost entirely unstructured in the native protein. A point of note, though, is that multiple phosphorylation sites have been mapped within this sequence: Phosida (Olsen *et al*. 2006) annotates 10 sites within our 29mer that are phosphorylated *in vivo*. In the future, it will be of great interest to establish if spectrin-CAMSAP1 interaction is modulated by phosphorylation.

Since the sequence LEEK is characteristic of the CC1 region, and highly conserved in evolution (Fig. 1b and c), we tested a possible significance of this sequence *in vitro* (spectrin binding, Fig. 2d) and in living PC12 cells (Table 1, Fig. 5). Mutation of LEEK to AAAA inhibits binding of spectrin to CAMSAP1 peptide *in vitro*. Over-expressed CC1 is partially inhibitory to neurite outgrowth (Table 1), and the AAAA mutation stops this. The AAAA mutant is also incapable of fully rescuing the siRNA knockdown. These data point to a link between CAMSAP1-spectrin interaction and the process of NGF-induced neurite outgrowth. It is also of note that peptides CC1-P1 and CC1-P2 both contain the LEEK sequence, but only CC1-P1 binds spectrin: this suggests that the requirements for spectrin binding extend beyond the LEEK sequence; LEEK, while necessary, is clearly not sufficient for interaction.

The CC1 region also bound to Ca^2+^/calmodulin (Fig. 3), a protein known to be closely associated with the control of axon extension and guidance. In PC12 cells, calmodulin has a central role in differentiation of the cells and production of neurites (Davidkova *et al*. 1996; Hoshino and Nakamura 2003). Since both spectrin and calmodulin bound to CC1, we tested of they compete with each other for binding – as indicated in Fig. 3, this appears to be the case. Interestingly, although the spectrin 29mer and Ca^2+^/calmodulin bound to CC1 with similar affinities, activation of calmodulin in brain extract was able to completely block immunoprecipitation of spectrin and CAMSAP1: presumably this reflects the high concentration of calmodulin in brain. It is worthy of note that the gene encoding CAMSAP1 is controlled by the transcription factor calcium-response factor (West 2011). In future work it will be of great interest to establish the control of both CAMSAP1 expression and functional interplay with binding partners such as spectrin, to identify the nature of its role in calcium signalling.

As indicated in Figs 4 and 5, siRNA knockdown of CAMSAP1 results in a lack of neurite production by PC12 cells in response to NGF. Interestingly, the effect of CAMSAP1 loss on the PC12 cells did not appear to result from lack of sensitivity to NGF. Over several days of culture, the cells became bigger than controls both in the diameters of cell bodies and their nuclei (Fig. 4c). This might suggest that the cells are responding to NGF by enhancing protein synthesis, but they are unable to direct the resulting material into the production of extensions to the cell body.

Mutation of CC1 at the LEEK sequence abolished spectrin interaction *in vitro*, and mutant CAMSAP1-EGFP construct gave reduced rescue of the siRNA knockdown (Fig. 5): these data link CC1-spectrin interaction to neurite production. The mechanisms underlying the function of spectrin in axon/neurite outgrowth are still not fully resolved. In *C. elegans*, spectrin is dispensable for the initiation of axon outgrowth, but it is clearly required for the mechanical stability of axons: in the absence of spectrin, axons fractured under the strain of the movement of the animal (Hammarlund *et al*. 2007). A pointer to a more fundamental role in mammals comes from experiments probing the role of the spectrin-binding protein ankyrin (Nishimura *et al*. 2003). Although the role of spectrin was not tested per se, mutations in ankyrin that block spectrin binding inhibited axon outgrowth in response to a permissive external cue. In the βII-spectrin knockout mouse, the mice die *in utero* of major defects in the formation of mutiple organs, including the brain: this has been attributed to loss of signalling through the TGFβ [transforming growth factor (TGF)] pathway (Tang *et al*. 2003). αII-spectrin, the major brain partner of βII-spectrin, modulates the function of neural cell adhesion molecule (NCAM) during axon outgrowth (Ramser *et al*. 2010). Axon formation is impaired in the αII-spectrin knockout mouse (Stankewich *et al*. 2011).

A final consideration is that while the LEEK motif is present in CAMSAP1 throughout the eumetazoa (with the exception of acoelomates) (Fig. 1), the region of spectrin to which it binds (29 amino acids between the last triple helical repeat and the PH domain) is less conserved. Vertebrate βII-spectrin is a descendent of an ancestral invertebrate β-spectrin which underwent two duplication events during vertebrate evolution (Baines 2010a). Blast analysis (data not shown) suggests that the 29 amino acids of βII-spectrin that bind CAMSAP1 CC1 are characteristic of vertebrates, and may represent a specific adaptation of βII-spectrin. The strong conservation of the LEEK motif in invertebrate CAMSAPs potentially indicates further, as yet unidentified, functions for that motif.

## Abbreviations

CC1: CAMSAP-conserved region 1
EGFP: enhanced green fluorescent protein
NGF: nerve growth factor

## Supporting information

Table S1 Sequences used in Fig. 1.

## References

[b1] Ahmad FJ, Hughey J, Wittmann T, Hyman A, Greaser M, Baas PW (2000). Motor proteins regulate force interactions between microtubules and microfilaments in the axon. Nat. Cell Biol.

[b2] Altschul SF, Madden TL, Schaffer AA, Zhang J, Zhang Z, Miller W, Lipman DJ (1997). Gapped BLAST and PSI-BLAST: a new generation of protein database search programs. Nucleic Acids Res.

[b3] Baines AJ (2010a). Evolution of the spectrin-based membrane skeleton. Transfus. Clin. Biol.

[b4] Baines AJ (2010b). The spectrin-ankyrin-4.1-adducin membrane skeleton: adapting eukaryotic cells to the demands of animal life. Protoplasma.

[b5] Baines AJ, Bignone PA, King MD, Maggs AM, Bennett PM, Pinder JC, Phillips GW (2009). The CKK domain (DUF1781) binds microtubules and defines the CAMSAP/ssp4 family of animal proteins. Mol. Biol. Evol.

[b6] Bairoch A, Apweiler R, Wu CH (2005). The Universal Protein Resource (UniProt). Nucleic Acids Res.

[b7] Bennett V, Baines AJ, Davis J (1986). Purification of brain analogs of red blood cell membrane skeletal proteins: ankyrin, protein 4.1 (synapsin), spectrin, and spectrin subunits. Methods Enzymol.

[b8] Bignone PA, King MDA, Pinder JC, Baines AJ (2007). Phosphorylation of a threonine unique to the short C-terminal isoform of beta II-spectrin links regulation of alpha-beta spectrin interaction to neuritogenesis. J. Biol. Chem.

[b9] Davidkova G, Zhang SP, Nichols RA, Weiss B (1996). Reduced level of calmodulin in PC12 cells induced by stable expression of calmodulin antisense RNA inhibits cell proliferation and induces neurite outgrowth. Neuroscience.

[b10] Dedman JR, Kaetzel MA (1983). Calmodulin purification and fluorescent labeling. Methods Enzymol.

[b11] Dehmelt L, Halpain S (2004). Actin and microtubules in neurite initiation: Are MAPs the missing link?. J. Neurobiol.

[b12] Eddy SR (2011). Accelerated profile HMM searches. PLoS Comput Biol.

[b13] Goodwin SS, Vale RD (2010). Patronin Regulates the Microtubule Network by Protecting Microtubule Minus Ends. Cell.

[b14] Gordon-Weeks PR (2004). Microtubules and growth cone function. J. Neurobiol.

[b15] Goshima G, Wollman R, Goodwin SS, Zhang N, Scholey JM, Vale RD, Stuurman N (2007). Genes required for mitotic spindle assembly in Drosophila S2 cells. Science.

[b16] Guo YL, Baum LW, Sham PC (2012). Two-stage genome-wide association study identifies variants in CAMSAP1L1 as susceptibility loci for epilepsy in Chinese. Hum. Mol. Genet.

[b17] Hammarlund M, Jorgensen EM, Bastiani MJ (2007). Axons break in animals lacking beta-spectrin. J. Cell Biol.

[b18] Hayes NV, Phillips GW, Carden MJ, Baines AJ (1997). Definition of a sequence unique in beta II spectrin required for its axon-specific interaction with fodaxin (A60). J. Neurochem.

[b19] Hayes NV, Scott C, Heerkens E, Ohanian V, Maggs AM, Pinder JC, Kordeli E, Baines AJ (2000). Identification of a novel C-terminal variant of beta II spectrin: two isoforms of beta II spectrin have distinct intracellular locations and activities. J. Cell Sci.

[b20] Hirokawa N, Takemura R (2004). Kinesin superfamily proteins and their various functions and dynamics. Exp. Cell Res.

[b21] Hoshino M, Nakamura S (2003). Small GTPase Rin induces neurite outgrowth through Rac/Cdc42 and calmodulin in PC12 cells. J. Cell Biol.

[b22] Laemmli UK (1970). Cleavage of structural proteins during the assembly of the head of bacteriophage T4. Nature.

[b23] Lafont F, Rouget M, Rousselet A, Valenza C, Prochiantz A (1993). Specific responses of axons and dendrites to cytoskeleton perturbations: an in vitro study. J. Cell Sci.

[b24] Mangeat PH, Burridge K (1983). Binding of HeLa spectrin to a specific HeLa membrane fraction. Cell Motil.

[b25] McGuffin LJ, Bryson K, Jones DT (2000). The PSIPRED protein structure prediction server. Bioinformatics.

[b26] Meng W, Mushika Y, Ichii T, Takeichi M (2008). Anchorage of microtubule minus ends to adherens junctions regulates epithelial cell-cell contacts. Cell.

[b27] Nicol S, Rahman D, Baines AJ (1997). Ca^2^ ^+^ -dependent interaction with calmodulin is conserved in the synapsin family: Identification of a high-affinity site. Biochemistry.

[b28] Nishimura K, Yoshihara F, Tojima T, Ooashi N, Yoon W, Mikoshiba K, Bennett V, Kamiguchi H (2003). L1-dependent neuritogenesis involves ankyrinB that mediates L1-CAM coupling with retrograde actin flow. J. Cell Biol.

[b29] Olsen JV, Blagoev B, Gnad F, Macek B, Kumar C, Mortensen P, Mann M (2006). Global, in vivo, and site-specific phosphorylation dynamics in signaling networks. Cell.

[b30] Ramser EM, Buck F, Schachner M, Tilling T (2010). Binding of alphaII spectrin to 14-3-3beta is involved in NCAM-dependent neurite outgrowth. Mol. Cell. Neurosci.

[b31] Rice P, Longden I, Bleasby A (2000). EMBOSS: the European Molecular Biology Open Software Suite. Trends Genet.

[b32] Sayers EW, Barrett T, Benson DA (2009). Database resources of the National Center for Biotechnology Information. Nucleic Acids Res.

[b33] Scott C, Keating L, Bellamy M, Baines AJ (2001). Protein 4.1 in forebrain postsynaptic density preparations: enrichment of 4.1 gene products and detection of 4.1R binding proteins. Eur. J. Biochem.

[b34] Stankewich MC, Cianci CD, Stabach PR, Ji L, Nath A, Morrow JS (2011). Cell organization, growth, and neural and cardiac development require alphaII-spectrin. J. Cell Sci.

[b35] Tang Y, Katuri V, Dillner A, Mishra B, Deng CX, Mishra L (2003). Disruption of transforming growth factor-beta signaling in ELF beta- spectrin-deficient mice. Science.

[b36] Ward JJ, McGuffin LJ, Bryson K, Buxton BF, Jones DT (2004). The DISOPRED server for the prediction of protein disorder. Bioinformatics.

[b37] West AE (2011). Biological functions and transcriptional targets of CaRF in neurons. Cell Calcium.

[b38] Xu J, Ziemnicka D, Merz GS, Kotula L (2000). Human spectrin Src homology 3 domain binding protein 1 regulates macropinocytosis in NIH 3T3 cells. J. Cell Sci.

[b39] Yamamoto M, Yoshimura K, Kitada M (2009). A new monoclonal antibody, A3B10, specific for astrocyte-lineage cells recognizes calmodulin-regulated spectrin-associated protein 1 (Camsap1). J. Neurosci. Res.

[b40] Yap KL, Kim J, Truong K, Sherman M, Yuan T, Ikura M (2000). Calmodulin target database. J. Struct. Funct. Genomics.

