# Supplementary material for: A conserved sequence in calmodulin regulated spectrin-associated protein 1 links its interaction with spectrin and calmodulin to neurite outgrowth
Source: J Neurochem. 2013 Oct 24;128(3):391–402. doi: 10.1111/jnc.12462 (PMC4016758; doi:10.1111/jnc.12462)
Supplement: Table S1 — Sequences used in Fig. 1. [file jnc0128-0391-sd1.docx]

**Table 1S. Sequences used in Fig 1.**

| **Animal** | **Database** | **Accession** | **Organism** |
| --- | --- | --- | --- |
| Ant | Uniprot | F4WV87_ACREC | *Acromyrmex echinatior* |
| *Branchiostoma* | Refseq | XP_002588368.1 | *Branchiostoma floridae* |
| *C. Elegans* | Refseq | NP_510751.3 | *Caenorhabditis elegans* |
| *Capitella* | jgi\|Capca1 | fgenesh1_pg.C_scaffold_179000014 | *Capitella teleta* |
| Chicken 1 | Refseq | XP_415410.3 | *Gallus gallus* |
| Chicken 2 | Refseq | XP_422188.3 | *Gallus gallus* |
| Frog 1 | Refseq | NP_001084957.1 | *Xenopus laevis* |
| Frog 2 | Refseq | XP_002932197.1 | *Xenopus (Silurana) tropicalis* |
| Frog 3 | Refseq | XP_002941532.1 | *Xenopus (Silurana) tropicalis* |
| Fruitfly | Uniprot | SSP4_DROME | *Drosophila melanogaster* |
| Human 1 | Refseq | NP_056262.3 | *Homo sapiens* |
| Human 2 | Refseq | NP_982284.1 | *Homo sapiens* |
| Human 3 | Refseq | NP_001073898.1 | *Homo sapiens* |
| Hydra | Refseq | XP_002168541.1 | *Hydra magnipapillata* |
| Lizard 1 | Refseq | XP_003228392.1 | *Anolis carolinensis* |
| Lizard 2 | Refseq | XP_003223659.1 | *Anolis carolinensis* |
| Lizard 3 | Refseq | XP_003217191.1 | *Anolis carolinensis* |
| Lottia | jgi\|Lotgi1 | fgenesh2_pg.C_sca_173000028 | *Lottia gigantea* |
| Rat 1 | Refseq | NP_001162021.1 | *Rattus norvegicus* |
| Rat 2 | Refseq | NP_001127975.1 | *Rattus norvegicus* |
| Rat 3 | Refseq | NP_001138312.1 | *Rattus norvegicus* |
| Sea anemone | Refseq | XP_001620038.1 | *Nematostella vectensis* |
| Trichina | Uniprot | E5SIX6_TRISP | *Trichinella spiralis* |
| Sea urchin | Refseq | XP_001180210.1 | *Strongylocentrotus purpuratus* |
| Zebrafish 1a | Refseq | NP_001159727.1 | *Danio rerio* |
| Zebrafish 1b | Refseq | NP_001093471.1 | *Danio rerio* |
| Zebrafish 2a | Refseq | NP_001038461.1 | *Danio rerio* |
| Zebrafish 2b | Refseq | XP_003197845.1 | *Danio rerio* |
| Zebrafish 3 | Refseq | XP_684101.5 | *Danio rerio* |
